# On the Origin of the Vapour Bath

**Published:** 1829-03

**Authors:** Thomas Ridgway

**Affiliations:** Licentiate of the Royal College of Physicians, &c.


### VAPOUR BATH.

*On the Origin of the Vapour Bath.* By Thomas Ridgway, m.d.
Licentiate of the Royal College of Physicians, &c.

Looking over the whole range of medical remedies, we shall probably not find any one class of them so generally applicable and useful as bathing, or the immersion and ablution of the whole body, or of any part thereof, in a fluid medium, of a composition or temperature adapted to the particular end in view: and this, whether it be considered as a means of cleanliness, or as an adjuvant in the cure of disease.

The almost total neglect of this remedy in all its various forms in this country, has long been a subject of lamentation to those who were conscious of its value, and of the benefits which would necessarily arise from its universal adoption. As an elegant luxury, it is still known only to the few; as a means of cleanliness, it is almost totally neglected; and the medical practitioner but rarely avails himself of its powerful influence and assistance.

Although the simple immersion and ablution of the body in water is attended with great advantages, in preserving cleanliness, promoting health, and removing disease, there can be no doubt that, as a remedy, it may be rendered much more powerful, and, therefore, in many instances more efficient, by elevating the temperature of the fluid so as to raise it into vapour, involving the whole body in a warm exhalation, mollifying, and disposing the adherent recrementitious matters to be thrown off; while, by the increased heat, the fluids of the body are invited from the centre of the system towards its circumference, and permitted to pour themselves forth in profusion over its infinitely porous and permeable surface.

The physicians of Paris have, of late years, most meritoriously turned their attention to the perfection of this form of immersion, termed the vapour bath, which, both in its simple and its medicated shape, has been productive of incalculable advantage. Mr. Green, an intelligent surgeon, formerly of the royal navy, has, with much pains and assiduity, introduced these baths into England, and established them upon an extensive and admirable plan in a central situation in London, so that medical practitioners have the advantage of directing them in any mode they may think proper, under a superintendence with which they cannot but feel satisfied.

It is, however, curious to know that, notwithstanding we seem now to have derived this method of bathing from France, it was originally invented in this country. There has lately fallen into my hands a book, wherein both the general vapour bath, as well as that called by the French *douche*, or stream bath, are both of them accurately described; and that even under, at least an attempt towards, a medicated form. The book is entitled "A Physico-Medical Essay concerning Alkali and Acid, so far as they have relation to the Cause or Cure of Distempers, &c. By John Colbatch, Physician. London: printed for Dan. Browne, at the Black Swan, without Temple Bar. MDCXCVI." That is, about 130 years ago; and the passage which alludes to our present subject is as follows:

"Besides the service done to mankind by drinking of mineral waters, what advantage does accrue to many people labouring under some sort of nervous distempers, &c. by merely bathing themselves in the nitro-sulphureous hot baths.

"There is also a new way of sweating, by the means of the volatile-acid steams arising from the evaporating brine, in the making of salt at our English salt-pits, lately invented by Mr. Henry Hodges, of Droyt-Wych in Worcestershire; by the means of which several very great things have been done, even in cases where the Bath, common hummums, and bagnios, have proved altogether ineffectual. I am sorry that I am at a place where I cannot procure a number of experiments to insert in this place, which might be of service to mankind; but, to supply the place of them, I shall add something done in a little bathing-house I erected of my own, wherein I imitated, if not outdid, the way of sweating at Droyt-Wych; but my many avocations hindered me from the prosecuting of it.

"I procured a quantity of the virgin salt from the salt rock in Cheshire, and, as I had occasion, I dissolved a convenient quantity of it in spring water, making a brine as strong as that obtainable from the brine-pits. With this brine I filled a large iron pot, which had pipes of wood went from it to a little room overhead, made convenient for people to sweat in. Under my pot I made a fire, which both warmed the room and made the brine to boyl, and from the boyling brine arose such quantities of steams as filled my room, which, when it was warmed and full of steams, was fit for use. I had, besides the large pipes which supplied the whole room, several others of different lengths, by the means of which I more forcibly conveyed the steams to any particular part. By this way of sweating, I have known a gentlewoman cured, as was also one at Droyt-Wych, of an inveterate leprosy, which had eluded the efficacy of all other medicines and baths. It rarely failed taking off the most violent old aches and pains. In all relaxations of the nerves and tendons, I have never met with any thing comparable to it. To be short, I found it as good as the Bath in most things, and in many out-did it; and I believe Mr. Hodges, computing the time he has used the way of sweating at his brine-pits, and the number of people he has had, can produce a greater catalogue, and more considerable cures wrought, than hath been at the Bath."

To those who have had the good fortune to visit the Hospital of St. Louis at Paris, the above description cannot fail to bring forcibly to remembrance the internal arrangements of that fine institution; the sudatory, with its numerous orifices pouring forth vapour on the persons of those arranged within it, and the streams and jets directed to any particular part.

To the French physicians belongs, in all probability, the merit of reviving the use of the vapour bath; certainly of bringing it, together with a knowledge of the diseases of the skin, to a degree of perfection which, without their efforts, would scarcely have been attained. With us it remains to reflect that a knowledge of this admirable remedy was first elicited among ourselves; to be sensible how long and injuriously we have neglected it; and to make now the atonement which is alone in our power, of urging ourselves and others to its more frequent adoption, and of bestowing that attention on its properties, whereby we may make it to approach nearer to perfection, and become more extensively beneficial.

*London; January 22d,* 1829.

---

